# Standard Versus Enhanced Measurement-Based Care Effectiveness for Depression (EMBED): Protocol for a Cluster Randomized Implementation-Effectiveness Trial

**DOI:** 10.7759/cureus.90395

**Published:** 2025-08-18

**Authors:** Raymond W Lam, Erin E Michalak, Jill K Murphy, Heather Colquhoun, Chee Ng, Larry Culpepper, Carolyn S Dewa, Andrew J Greenshaw, Yanling He, Sidney H Kennedy, Xinmin Li, Jing Liu, Tianli Liu, Sagar Parikh, Claudio N Soares, Zuowei Wang, Yifeng Xu, Jun Chen

**Affiliations:** 1 Psychiatry, University of British Columbia, Vancouver, CAN; 2 Health, St. Francis Xavier University, Antigonish, CAN; 3 Rehabilitation Medicine, University of Toronto, Toronto, CAN; 4 Department of Psychiatry, University of Melbourne, Melbourne, AUS; 5 Family and Community Medicine, Boston University, Boston, USA; 6 Psychiatry and Behavioral Sciences, University of California Davis, Davis, USA; 7 Psychiatry, University of Alberta, Edmonton, CAN; 8 Psychiatry, Shanghai Mental Health Center, Shanghai, CHN; 9 Psychiatry, University of Toronto, Toronto, CAN; 10 Institute of Population Health, Peking University, Beijing, CHN; 11 Psychiatry, University of Michigan, Ann Arbor, USA; 12 Psychiatry, Queen's University, Kingston, CAN; 13 Psychiatry, Hongkou Mental Health Centre, Shanghai, CHN

**Keywords:** assessment, cluster trial, depression, implementation, measurement-based care, mixed-methods, mobile health, randomized, scales, wechat

## Abstract

Background: Measurement-based care (MBC) is an evidence-based practice that incorporates routine outcome assessment using validated rating scales to guide collaborative clinical decision-making. Although MBC results in improved outcomes for patients with major depressive disorder (MDD), there are barriers to its broad implementation in clinical settings. The use of “enhanced” MBC (eMBC), with mobile apps that allow patients to track outcomes and engage in self-management via WeChat, may address some of these barriers. We hypothesize that implementation with eMBC using WeChat will be superior to standard MBC implementation using paper-pencil assessments at the clinic, for both implementation and clinical outcomes.

Methods: We present a trial protocol (clinicaltrials.gov NCT05527951) for a two-arm cluster randomized clinical trial (RCT) with a hybrid implementation-effectiveness design comparing standard MBC implementation versus eMBC implementation with a six-month follow-up in 12 mental health centers in Shanghai, China. The eMBC implementation uses a WeChat mini-program that includes outcome tracking using brief questionnaires and self-management lessons supplemented with support by a lay coach via WeChat.

Results: A total of 240 physicians and 1200 patients from the 12 mental health centers will be enrolled in the mixed-methods outcome analysis. The primary implementation outcome is *implementation reach*, defined as the proportion of eligible patients with a PHQ-9 score recorded in the hospital chart at six months after MBC implementation. The primary clinical outcome is *clinical remission*, defined as a PHQ-9 score of 4 or less at the six-month follow-up. Other implementation and clinical outcomes will be examined, including medication adherence, doctor-patient alliance, and a piggy-back cost-benefit economic analysis. Qualitative interviews will be conducted with physicians and patients to produce an interpretive account of the contextual factors that impact eMBC implementation.

Conclusions: The results of this hybrid implementation-effectiveness cluster RCT will inform implementation of eMBC with WeChat mobile apps for patients with depression in other clinical settings in China and internationally.

## Introduction

Major depressive disorder (MDD) affects more than 300 million people globally and is now the leading cause of disability and burden of disease worldwide [1]. Across the 36 largest countries in the world, more than 50 million years of lost productivity are attributable to depression and anxiety disorders every year, at an estimated cost of $925 billion [2]. On the other hand, scaled-up treatment of MDD can lead to large economic productivity gains of a net value of $230 billion. Despite known effective treatments for depression, fewer than half of those affected in the world (in many countries, fewer than 10%) receive such treatments [1]. Barriers to effective care include a lack of human and service resources, social stigma, and limited access to proper assessment, outcome measurement and monitoring, and evidence-based treatments, especially psychological treatments.

In China, depression is the second-most diagnosed disease (after heart disease) and costs the nation an estimated 52 billion yuan ($8.35 billion) every year [3]. China’s mental health care resources have developed quickly during the past three decades, but more growth is needed [4]. Most of the mental health resources, including psychiatrists and psychiatric inpatient beds, are concentrated within the larger cities, leaving the vast suburban and rural regions with few services for mental health treatment, and most psychiatric services in China still primarily serve patients with psychotic disorders. Yet, there are also indicators to suggest that China is primed to scale up in evidence-based practices. Many general hospitals have begun offering mental health services for patients with common nonpsychotic mood disorders such as MDD. Chinese guidelines for the treatment of MDD have been recently revised and the National Mental Health Work Plan 2015-2020 specifies improving the ability of healthcare facilities to identify depression and increasing the treatment rate by 50% [5].

Measurement-based care (MBC) - the routine use of simple, validated outcome scales such as the Patient Health Questionnaire (PHQ-9) depression scale [6] to guide clinical decision-making in treating MDD - is an evidence-based practice that may help meet these ambitious objectives [7]. The self-rated PHQ-9 can be helpful in screening, diagnosis and monitoring treatment; in primary care and other settings, the PHQ-9 can increase the recognition of MDD and improve outcomes when used within an MBC management approach [7]. For example, simple algorithms based on PHQ-9 scores can help physicians decide when to change the dose of an antidepressant or when to switch to another [7,8]. Importantly, it can help identify which patients are not improving so that more intensive treatment can be offered. MBC is shown to improve both clinical outcomes [9] and patient adherence to treatment [10], including in studies conducted in China [11], as summarized by meta-analysis [12]. However, despite recent policy-level recommendations for broad implementation of MBC [13,14] and recommendations in clinical practice guidelines [15], there is still a significant care gap in the implementation of MBC - only a minority of physicians and clinicians (in some studies, only 18% of psychiatrists and 11% of psychologists) use MBC in their clinical practice [16].

Study rationale

Implementation science has shown that adoption of an evidence-based practice like MBC by physicians and healthcare professionals is affected by many factors at various levels of organization [17]. In Phase 1 of the Enhanced Measurement-Based care Effectiveness for Depression (EMBED) study, we conducted a Situational Analysis addressing organization, clinician, and patient levels to determine the key barriers and facilitators for MBC implementation in Shanghai [18]. We found that, consistent with the literature [19,20], barriers include perceived time constraints, not knowing how to use measurement in clinical decisions, and challenges with integrating measurement into clinic workflow. We also found many facilitators to MBC implementation including positive attitudes about the clinical utility of MBC, interest in training in the use of scales, and beliefs that MBC would increase patient empowerment and reduce stigma [18].

Using the results from the Shanghai situational analysis [18], we adapted a standard MBC implementation plan [21] within an evidence-based framework, the Behavioural Change Wheel, that addresses contextual factors at administrative, organizational, clinician and patient levels [22]. The key elements of the EMBED MBC implementation plan (Appendix A) include support of hospital administration, identification of champions, clear policies, mapping of clinic workflow, MBC training for clinic doctors and administrative staff, adapting charts or electronic medical records (EMRs) for monitoring, and regular monthly MBC team consultation meetings.

The concept of enhanced MBC (eMBC), in which mobile health (mHealth) tools and services can engage both patients and physicians in using MBC, may also offer solutions to some of the identified barriers [7,23]. These enhancements include online or mobile apps for patients to monitor and track progress using validated self-rating scales such as the PHQ-9. The results from these app-based tools can then be shared with their physicians (e.g., by showing them the tracking charts or printing out the scores), thereby promoting a collaborative approach to MBC. The apps also have reminder systems (in-app notifications on mobile devices) so patients can be reminded to complete their questionnaires regularly (e.g., every two to four weeks) with messages to return to their physician (or other health provider) if their depression is not improving.

Enhanced MBC can also include interventional features such as Bounce Back, an evidence-based depression self-management [24] program delivered in Canada by the Canadian Mental Health Association [25]. Bounce Back was originally developed by Dr. Chris Williams in the United Kingdom as primarily a self-help book supported by brief guided support [26]. Bounce Back consists of an online self-directed program based on cognitive-behavioural therapy strategies, supplemented with telephone support from a lay coach [25].

We have extended the concept of technology-enhanced mood measurement with the use of WeChat. WeChat is a Chinese multi-purpose messaging, social media and mobile payment app developed by Tencent that has become the world's largest standalone mobile app, with over 1 billion monthly active users in 2018. WeChat has been described as China's “app for everything” which provides text chats, video and audio conferencing, games, photo and video sharing, and other features [27]. WeChat Mini Programs are “mini-applications” built within the WeChat platform, developed by third-party companies to provide advanced features to users that can run within the app [28]. We have created a WeChat mini-program, which includes mood tracking and self-management lessons (similar to Bounce Back), to support eMBC.

In EMBED Phase 2, we will evaluate eMBC implementation using our WeChat mini-program by conducting a cluster randomized clinical trial (RCT) to compare the effectiveness of eMBC implementation versus standard MBC implementation in a real-world setting, i.e., mental health centres in Shanghai. The specific objectives for Phase 2 include: (1) To determine the incremental effect of eMBC over standard MBC on implementation and fidelity outcomes; (2) To determine the incremental effect of eMBC over standard MBC on clinical, health service and economic outcomes, and (3) To understand the contextual physician and patient mediators that affect eMBC implementation. We hypothesize that eMBC will outperform standard MBC on implementation, clinical, and health economic outcomes at both physician and patient levels.

A previous version of this article was posted at the Open Science Framework preprint server on August 26, 2021 (https://doi.org/10.31219/osf.io/9z82u).

## Materials and methods

This cluster-randomized trial is registered at www.clinicaltrials.gov [NCT05527951] in accordance with the policy from the International Committee of Medical Journal Editors [29]. The study protocol involves a two-arm cluster RCT with a hybrid implementation-effectiveness design [30] with a six-month follow-up. Using this design, we can compare the incremental benefit of eMBC over standard MBC for both implementation outcomes as well as clinical and health services outcomes.

**Figure 1:**
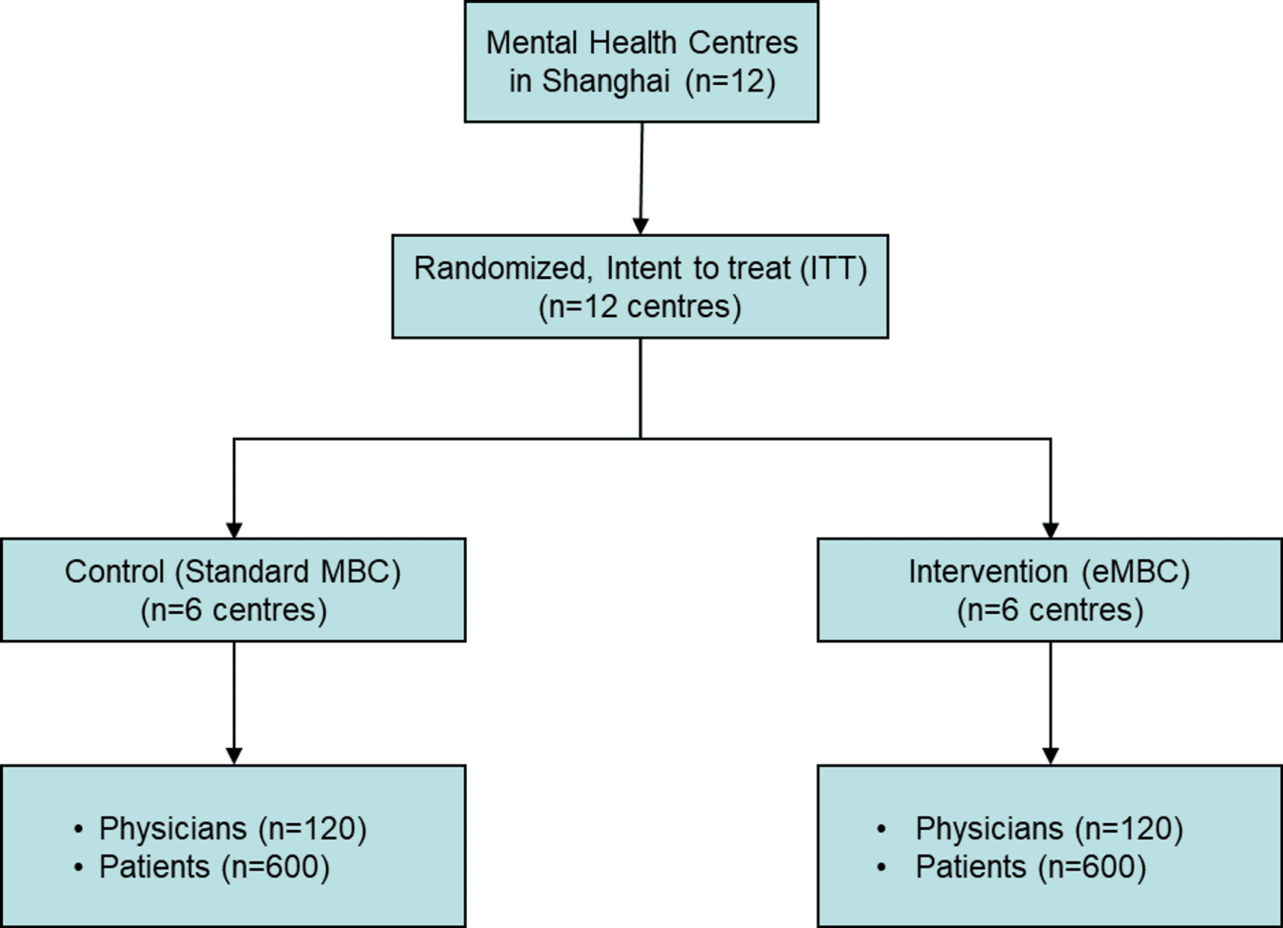
CONSORT diagram CONSORT, Consolidated Standards of Reporting Trials; MBC, measurement-based care; eMBC, enhanced measurement-based care.

We randomly allocated 12 mental health centres in Shanghai to two intervention groups (Figure 1). The unit of randomization was the mental health centre. We paired an urban centre with a rural centre to adjust for population size and to ensure a balance of urban and rural centres. A statistician blind to identity of the centres used a computerized program to randomly allocate each pair to control and experimental interventions in a 1:1 ratio. The centres allocated to the control intervention group will implement standard MBC using paper and pencil questionnaires [21] while those allocated to the experimental group will implement eMBC with our WeChat mini-program, *Easy to Recover from Depression*, which consists of mood tracking and lay-coached self-management (see Interventions). Once randomized, clinic centre staff and project personnel will not be masked to intervention assignment. Demographic and clinical measures will be collected from all participants via another WeChat mini-program called *eSmile*. The primary implementation outcome is Implementation Reach at six months, defined as proportion of eligible patients with a PHQ-9 score recorded in the chart or EMR, based on chart review. The primary clinical outcome is PHQ-9 remission (score ≤4) at six-month follow-up.

Study design

We anticipate implementation in these 12 clinics with 240 physicians (about 20 per clinic). We will enroll 1200 eligible patients (about 100 per clinic) to assess clinical outcomes. The clinics allocated to standard MBC implementation will follow the implementation plan designed from EMBED Phase 1 (Appendix A). The implementation plan includes a package of validated outcome scales, standardized guidelines and algorithms for depression treatment based on adaptation of Chinese and Canadian Network for Mood and Anxiety Treatments (CANMAT) guidelines for MDD [15], MBC training workshops/webinars for physicians and staff, and monthly implementation meetings during the six months of follow-up. Clinics randomized to eMBC implementation will also be trained on the use of the *Easy to Recover from Depression* WeChat mini-program.

Study population

Study Sites

Of the 22 Shanghai community mental health centres, we identified 12 participating outpatient clinics from the Phase 1 situational analysis from the Hongkou Mental Health Centre, Fengxian Mental Health Centre, Songjiang Mental Health Centre, Xuhui Mental Health Centre, Changning Mental Health Centre, Nanhui Mental Health Centre, Yangpu Mental Health Centre, Qingpu Mental Health Centre, Pudong Mental Health Centre, Baoshan Mental Health Centre, Huangpu Mental Health Centre, and Putuo Mental Health Centre. These sites represent urban, suburban and rural regions of Shanghai. The Shanghai Mental Health Center will be the coordinating site for the study. Recruitment started at the Hongkou and Fengxian sites in January, 2023, with final site study visits anticipated to be completed in December, 2026.

Participants - Physicians

All doctors (n=20 per clinic, 240 total) working at each clinic will be involved in the MBC implementation. A sample of doctors (n=24) will be randomly selected for the qualitative interview assessments.

Participants - Patients

Because this is a pragmatic evaluation of an evidence-based practice, the eligibility criteria will be inclusive to ensure a broad representation of patients: (1) age 18 years and older; (2) diagnosis of unipolar depressive disorder made by the treating physician based on accepted diagnostic criteria (e.g., Chinese Classification of Mental Disorders (CCMD)-3, Diagnostic and Statistical Manual of Mental Disorders (DSM)-IV, DSM-5, International Statistical Classification of Diseases and Related Health Problems, 10th Revision (ICD-10)). A sample of patients (n=100 per clinic, 1200 total) will be selected to complete comprehensive clinical assessments (see Outcomes) via a WeChat research outcomes mini-program (called *eSmile*), which is separate from the eMBC *Easy to Recover from Depression* WeChat mini-program.

Interventions

Standard MBC Implementation

The standard MBC implementation is based on the EMBED implementation plan developed from the Situational Analysis tailored to the Shanghai context (for details, see Appendix A). In summary, the chosen MBC outcome measures include four brief, validated self-rated scales: PHQ-9 for depressive symptoms, Generalized Anxiety Disorder scale (GAD-7) [31] for anxiety symptoms, Sheehan Disability Scale (SDS) [32] for functioning, and the Frequency, Intensity, Burden of Side Effects Rating (FIBSER) [33] for medication side effects. These scales have been validated in Chinese populations [34-37]. The implementation plan includes workflow mapping to embed the scales and monitoring forms into clinic charts and workflow, e.g., flagging charts for patients with depression so that they receive the paper scales after registering for their clinic appointment and completing the scales while in the waiting room. The doctors will receive standardized training on MBC, including the use of scales and monitoring forms, how to discuss scores with patients, and simple treatment algorithms based on outcome measurement. Administration and clinical champions will be identified to lead the implementation and regular meetings with a consultation team will be held to collaboratively problem-solve implementation challenges.

Enhanced MBC (eMBC) Implementation

The eMBC implementation includes all the elements of the standard MBC implementation plan described, but patients will use the WeChat mini-program, *Easy to Recover from Depression*, instead of paper-pencil questionnaires at the clinic. The doctors and staff of clinics allocated to eMBC implementation will receive additional training on the *Easy to Recover from Depression* WeChat mini-program, which consists of the following two components.

Mood Measure:* *The *Mood Measure* component of the *Easy to Recover from Depression* mini-program consists of four scales to assess the symptoms of depression, functioning, and side effects of medications: PHQ-9, GAD-7, SDS, FIBSER. *Mood Measure* features include graphical display of scores, explanation of scores, graphical tracking of recent scores, and Frequently Asked Questions with answers. A scheduled reminder feature allows patients to set reminder notifications to complete the scales and through Print/Share function they can print or share the results.

Come Back:* *The University of British Columbia (UBC) and Shanghai Mental Health Centre (SMHC) teams have translated and culturally adapted the Bounce Back program for use in China, to be delivered via WeChat. The *Come Back* component of the *Easy to Recover from Depression* mini-program consists of six weekly lessons based on cognitive-behavioural therapy (CBT) principles. Each lesson includes an introductory video, narrated slides, a workbook, and worksheets to be completed by the patient. The six depression lessons include: (1) Understanding Low Mood and Depression; (2) Practical Problem Solving; (3) Doing Things That Boost How You Feel; (4) Noticing Extreme and Unhelpful Thinking; (5) Changing Extreme and Unhelpful Thinking; and (6) Understanding and Using Antidepressants.

In addition, patients using *Come Back* can connect with a coach via WeChat or telephone. The coaches are lay people who are trained to provide support and encouragement to complete the lessons. Coaches are available to patients for up to 20 minutes per week for six total sessions via chat, audio, or video link, depending on patient preference. Coaches are supervised via WeChat videoconference by a clinical psychologist based at Shanghai Mental Health Center. Fidelity will be assessed by the supervising psychologist using chart notes.

Ethics, security, and confidentiality

Ethics and Informed Consent

The study is approved by the Ethics Committee at the Shanghai Mental Health Centre (2020-77C2) and the Clinical Research Ethics Board at the University of British Columbia (H21-02581). Because both interventions involve implementation of an evidence-based practice, no individual consents are required for patients at each mental health centre. However, the selected patients for evaluation of clinical outcomes will provide informed consent via the WeChat *eSmile* research outcomes mini-program, assisted by research assistants at each site. In addition, patients and doctors selected for qualitative interviews will provide written informed consent.

Data Security and Confidentiality

All study data will be collected in China. The UBC study team will only have access to de-identified data from the trial conducted in Shanghai. We will comply with Standard Operating Procedures for data safety, management, sharing, storage, and retention with reference to relevant legislation and guidelines in both Canada and China. Data will be stored in a secure site managed by the PI and study staff in China, and will be transferred to UBC through *UBC TeamShare*, a secure data sharing platform.

## Results

Outcomes

Enrollment in the study started at one site on September 15, 2022. We have completed enrolment in two study sites, are currently enrolling in four study sites, and will start enrolling in the other six sites in January, 2025. We anticipate that enrollment will be completed by December 31, 2025, final follow-up data will be collected by June 30, 2026, and results will be available by October, 2026.

This study will evaluate both implementation and clinical outcomes. The implementation outcomes (Table 1) are based on definitions proposed by Proctor et al. [38] and are assessed via chart/EMR review at six months after MBC implementation. The primary implementation outcome will be implementation ‘Reach,’ defined as the proportion of patients with a PHQ-9 score recorded in the chart or EMR.

**Table 1:** Implementation outcomes, definitions, and measures. EBP, evidence-based practice; MBC, measurement-based care; PHQ-9, Personal Health Questionnaire; EMR, electronic medical record.

Implementation outcome (alternate terms)	Definition (using EBP as the implementation)	Standard MBC Implementation (data source in brackets)	eMBC Implementation, in addition to those in standard MBC (data source in brackets)
Adoption (Uptake)	Initial action to try or employ an EBP	Proportion of physicians who record at least one PHQ-9 score in the chart (EMR/chart)	Proportion of patients who accessed the WeChat Easy to Recover from Depression mini-programs at least once (app, coach)
Penetration (Reach)	Integration of an EBP within a service setting and its subsystems	Proportion of patients with a PHQ-9 score recorded (EMR/chart)	Proportion of patients who accessed the WeChat Easy to Recover from Depression mini-programs at least twice (app); Proportion of patients who accessed at least 2 sessions of coaching (chart review, coach)
Fidelity (Adherence)	Degree to which an EBP was implemented as planned	Proportion of patients where a PHQ-9 score was used in a clinical decision (interviews)	Proportion of patients who completed 4 of 6 lessons in the Come Back program (app).
Acceptability (Satisfaction)	Perception of stakeholders that an EBP is agreeable or satisfactory	Evidence-Based Practice Attitude Scale (EBPAS) [39] (survey of physicians)	System Usability Scale [40] (survey of patients)
Appropriateness (Compatibility)	Perceived fit, relevance, or compatibility of the EBP	Qualitative exploration in physicians (interviews)	Qualitative exploration in patients (interviews)
Cost (Resources)	Cost impact of an EBP	Costs of MBC training program; estimated costs of workflow (based on Chinese sources)	Costs of eMBC elements, incl WeChat apps and coaching (based on Chinese sources)
Sustainability (Maintenance)	Sustained use and durability of an EBP	Qualitative exploration in physicians (interviews)	Qualitative exploration in patients (interviews)

The clinical outcomes are assessed at baseline, two months, four months, and six months (Table 2). The primary clinical outcome will be PHQ-9 remission rate (PHQ-9 score ≤ 4) at six months. Other clinical outcomes (scales) will include PHQ-9 change scores, anxiety symptom change scores (GAD-7), functional disability (SDS), quality of life (QOL-6 [41], EQ-5D-5L [42]), health economic assessment (HEA [43]), medical adherence (Patient Adherence Questionnaire, PAQ [44]) and doctor-patient alliance (Scale To Assess Therapeutic Relationships in Community Mental Health Care STAR-P, [45]).

**Table 2:** Clinical outcomes and measures.

Outcome	Patient-Rated Scale
Depressive symptoms	Personal Health Questionnaire (PHQ-9) [6]
Anxiety symptoms	Generalized Anxiety Disorder (GAD-7) [30]
Functioning	Sheehan Disability Scale (SDS) [32]
Quality of life	Quality of Life scale (QOL-6) [41]; EuroQoL 5 Dimensions (EQ-5D) [42]
Medication adherence	Patient Adherence Questionnaire (PAQ) [44]
Doctor-patient alliance	Scale To Assess Therapeutic Relationships in Community Mental Health Care (STAR-P) [45]
Health services utilization	Health Economics Assessment (HEA) [43]

These patient-rated outcomes and basic demographic information will be collected from all patient participants via *eSmile*, a separate WeChat research outcomes mini-program created for this study. The *eSmile *mini-program includes a consent notice with each survey. A reminder notification will be automatically sent out to remind patients to complete the scales at scheduled dates, with a follow-up reminder after 24 hours if scales are not completed.

Economic outcomes will include health service utilization data (both mental health- and non-mental health-related) from the HEA and from available EMR/chart records, including health provider encounters, emergency room visits, hospitalizations, diagnostic tests, and prescriptions.

Sample size and data analysis

Sample Size

For the clinical outcomes, we will select 1200 participating patients, representing about 100 patients per clinic and about five patients per physician. The sample size of 240 physicians and 1200 patients (based on 12 clinics and 100 patients per clinic) will allow us to detect a minimal effect size between conditions with odds ratios of about 1.3 for categorical outcomes such as Implementation Reach and PHQ-9 remission rate, and about d=0.25 for continuous outcomes such as Implementation Acceptability (EBPAS scores) and patient functionality (SDS scores). These are regarded as small but clinically relevant effects; for comparison, these are similar to effect sizes found for comparisons of antidepressant medications versus placebo.

Data Analysis

We will use intent-to-treat samples for the analysis of implementation and clinical outcomes. Prior to the final data analysis, a full statistical analysis plan will be published. In summary, the statistical approach for dichotomous variables (e.g., proportion of patients with a documented PHQ-9 score; PHQ-9 remission rates) will use mixed-effects logistic regression models and for continuous variables (e.g., PHQ-9 and SDS scores at two-, four-, and six-month assessments) will use mixed-effects regression models, with compound symmetry covariance matrices to account for clusters created by clinics and longitudinal data, and adjusting for relevant (and especially equity-sensitive) baseline variables including age, sex, baseline severity, etc. These models belong to the Generalized Linear Mixed-effects Models (GLMMs) that are the most efficient and recommended statistical methods for analyzing clustered and longitudinal clinical trial data [46]. GLMM can account for data missing at random without requiring imputations of the missing values [47]. These analyses will allow us to examine main effects of cluster (e.g., potential differences in care quality between sites) and interaction effects between the two assigned conditions. Results will be reported as adjusted odds ratios, effect sizes (e.g., Cohen’s d) and 95% confidence intervals.

This study design will also allow us to conduct a secondary “piggy back” economic analysis [48] using health service utilization data from the HEA and EMR/charts. For example, the economic evaluation can model the cost-effectiveness of eMBC compared to standard MBC by estimating the incremental net benefit (INB) using net benefit regression [49], thereby allowing the statistical methods to be extended to a cost-effectiveness setting. In addition to creating a cost-effectiveness acceptability curve (CEAC), we will determine the incremental cost-effectiveness ratio (ICER).

We will also conduct mediational analyses using structural equation modelling to explore the factors that mediate the impact of the intervention condition on both clinician-and patient-level outcomes. For example, we can examine mediation effects by using eMBC fidelity as the dependent variable, the intervention condition as the independent variable, and contextual factors identified in Phase 1 as mediating variables. We will follow recommendations for assessing mediation in a multilevel context [50] based on factors at clinic, physician, and patient levels. The sample size projected for the cluster randomized evaluation will allow us to detect mediation effect sizes of about κ2=0.15 for clinician and patient models, which represent medium effect sizes [50].

In addition, we will employ a ‘mixed-methods sequential explanatory design,’ an analytic lens that assigns priority to quantitative data findings and uses them to guide and inform qualitative phases [51]. Purposive sampling will identify sites that show low fidelity to eMBC for further qualitative exploration. As in Phase 1, we will use Interpretive Description methods in subsets of physicians and patients to explore their contextual and motivational factors for standard MBC and eMBC adoption as well as reasons for deviation. Regardless of the specific methods, sufficient data collection (e.g., interviews, focus groups) will occur at purposefully selected [52] diverse low-fidelity sites in Shanghai to produce a rich ‘interpretive account’ [53] of the contextual factors that impact eMBC implementation.

## Discussion

MBC is an evidence-based practice that improves outcomes in patients with depression, but implementation of MBC within clinical settings is still a challenge. Digital tools such as mobile apps represent both the present and the future for mental health care [54], especially in lower-resource countries where in-person care is not scalable to meet population needs [55]. Digital mood tracking tools may also support MBC [56] but there are also challenges for implementation of digital tools in routine clinical care [57]. Hence, an open question is whether enhanced MBC using digital tools can improve implementation over standard paper-pencil administration of scales. The results of this cluster RCT will help to answer that question by comparing the implementation of enhanced MBC, using the *Easy to Recover from Depression* mini-program in the WeChat digital platform, to standard MBC implementation. Encouraging preliminary data show that clinicians and patients seem interested and ready to adopt eMBC, as per surveys conducted in China and in Canada [17,58]. A pilot study also found some evidence that eMBC leads to improved clinical outcomes, such as change in the PHQ-9 [59].

Implementation of evidence-based practices requires a focused knowledge translation action plan [19]. The results of this implementation-clinical study will be presented at scientific conferences and published, using the CONSORT guidelines, in peer-reviewed scientific journals with open access availability. Results will also be communicated to clinicians, health service delivery policymakers, and the public via an integrated knowledge translation platform hosted by the Canadian Biomarker Integration Network in Depression (CAN-BIND, canbind.ca). These efforts will facilitate dissemination for both MBC and eMBC beyond the academic community.

Limitations of this study need to be considered. One limitation is that all the mental health centres are located in Shanghai, China, and hence the results may not be generalizable to health systems in other countries. Because masking of the intervention is not possible, there is a risk of cross-contamination of eMBC use by patients and physicians at sites allocated to standard MBC implementation, but this is mitigated by the fact that patients and physicians generally attend only one mental health centre. In addition, the eMBC *Easy to Recover from Depression* WeChat mini-program includes a self-management module so the effects of this intervention cannot be disentangled from the mood tracking features of standard MBC implementation. However, we consider the ability to incorporate a digital mental health intervention within eMBC to be one of the advantages of digitally-delivered MBC over standard MBC. The eMBC *Easy to Recover from Depression* mini-program is also developed specifically for the WeChat platform and would require adaptation, both cultural and digital, for use in other digital ecosystems. However, this would not be difficult to do and there is potential for much wider use of both components of the eMBC mini-program in other digital platforms. Finally, limited access to technology and problems with digital literacy may be barriers to the use of eMBC for some patients with depression.

## Conclusions

In conclusion, the increasing use of mobile and internet programs in medical treatment is transforming health care systems worldwide. The integration of mobile apps into an evidence-based practice like MBC (eMBC) not only has the potential to streamline the assessment process but also can facilitate greater accessibility and engagement for patients with depression. The EMBED cluster RCT will evaluate both implementation outcomes and clinical outcomes associated with eMBC delivered via WeChat. Positive results would support the implementation of eMBC through digital platforms, which could have significant benefit for depression management globally. Integrated knowledge translation activities can ensure that study results reach the target population of clinicians and health policy decision-makers. Finally, while recognizing potential barriers, eMBC as an evidence-based practice can help achieve the goal of providing better and more effective personalized treatment to individuals with depression.

## Appendices

Appendix A: summary of EMBED MBC implementation plan

A “standardized” method for MBC implementation was adapted from Lewis et al. [21]. The standardized method was conceptualized as a “best practices” approach to implementation, incorporating six discrete implementation strategies [60]: (1) a needs assessment; (2) workflow mapping and adaptation (e.g., embedding scales and monitoring forms into paper charts and electronic health records [EHRs] where available); (3) MBC training for doctors and clerical staff; (4) institutional guidelines and policies; (5) forming a team; and (6) regular consultation. A psychiatrist with MBC expertise and a research coordinator will lead the strategies and activities over a 1-month active implementation period, followed by a 6-month sustainment period.

1. Needs assessment

A brief needs assessment will be conducted in each clinic prior to implementation. The results will guide specific aspects of implementation, such as identifying clinic MBC champions and change agents.

2. Workflow process mapping and adaptation

The current workflow in the clinic will be mapped and adapted for MBC implementation. These adaptations will include:

· Identifying charts for patients with depression (e.g., with a sticker on paper charts and a label on EHRs)

· Having a package of scales (PHQ-9, GAD7, SDS, FIBSER) to be given to patients by the clinic clerical staff.

· Having an info sheet (or video) for patients explaining the use of scales and how to complete them.

· Having patients complete the scales in the waiting room while waiting to see the doctor.

· Having the doctor briefly review the scores (and score history) with patients.

· Having an MBC monitoring form placed in the chart or EHR so the doctor can enter in the scores.

· The tracking form will also record basic MBC fidelity data, such as whether scales were completed by patients, clinicians discussed scores with patients, whether scores were used in clinical decisions, and reasons why scores were not entered.

3. MBC training

Doctors complete 2 hours (approximately) of training over 1 month. This training will have three components:

(1) A “Grand Rounds,” presented by a senior clinician, about the background, rationale, and scientific evidence for MBC, and the clinic decision to adopt. The primary goal is to introduce clinicians to MBC and build foundational knowledge about the scales and algorithms.

(2) A “hands-on” workshop with goals to build skill regarding the three core components: (1) the work flow for how the scales will be administered in the clinic, (2) how to enter and review scores with the tracking form, and (3) how to discuss scores with the patient.

(3) A workshop with goals to build skills for algorithmic care using MBC, such as when to increase medication doses and when to switch medications, and clinical tips for addressing lack of progress.

The training workshops cover core components of MBC, individual scales, and clinical tips. Active learning strategies are used for training guided by adult learning principles [61]. The trainer (preferably a clinic doctor) provides didactic content, models how to administer the scales, engages clinicians in practicing MBC core components, offers immediate feedback, and shows videos on how to use MBC with challenging patients. Doctors are invited to ask questions and participate in group discussion. The workshops will be evaluated using standard evaluations. Doctors may earn CME credits for the workshops.

Clerical staff will be trained separately to implement workflow changes with the scales package and monitoring forms. A resource person (i.e., one of the research staff) will be available on-site at least one day a week to oversee workflow and problem solve difficulties. In addition, the resource person will be available by telephone/WeChat contact during working hours.

4. Institutional guidelines and policies for implementing MBC

The mental health centre administrators will adopt recommended policies to implement MBC and these will be communicated (by usual centre methods) and disseminated to clinicians and clinic staff during the initial training and throughout the consultation meetings. This guideline is informed by the available scientific and policy literature stating that doctors ought to administer, review, and discuss the scale scores with depressed adult patients during every session.

5. Building consultation teams

It is well-documented that training alone is insufficient for changing behavior, hence consultation is needed to increase skill and support implementation [61]. Regular team consultation meetings will be held to consolidate MBC skills. Local opinion leaders (i.e., individuals who hold influence in a social network) and champions (i.e., individuals who vocally support an innovation) will be identified (perhaps by self-report measures such as the Opinion Leadership Scale [62] during the needs assessment). The local opinion leaders and staff champions are especially encouraged to prioritize their attendance at the team consultation meetings, since they play a pivotal role in their clinic.

6. Consultation team meetings

The primary goal of the brief consultation meetings is to facilitate doctors’ use of MBC with fidelity to the core components. Each meeting is led by a local staff champion and an external consultant (one of psychiatrists on the research team) via videoconference. Consultation meetings are held monthly during the 6-month active implementation period. For each meeting, two doctors may present examples of patients, either those with good outcomes, or those whom they tried but struggled to implement MBC. The team can then brainstorm solutions. Training booster sessions may also be held during the consultation meetings.

A brief, standardized case consultation form is completed prior to the meeting to provide the external consultant with context and specific questions. Active learning strategies (e.g., group discussion, modeling, practice + feedback) are used to engage doctors. Doctors may receive CME credits for each meeting.
